# Comparison of pigtail suture stent vs conventional DJ stent in ureteral stent symptom occurrences: A systematic review and meta-analysis

**DOI:** 10.1080/20905998.2025.2550798

**Published:** 2025-09-11

**Authors:** Krisna Adhitya Wilantara Yusuf, Ginanda Putra Siregar, Syah Mirsya Warli, Bungaran Sihombing, Dhirajaya Dharma Kadar, Yacobda H. Sigumonrong, Ramlan Nasution, Fauriski Febrian Prapiska, Kharisma Prasetya Adhyatma, Dianita Halimah Harahap, Muhammad Haritsyah Warli

**Affiliations:** a Department of Urology, Faculty of Medicine, Universitas Indonesia – Haji Adam Malik General Hospital, Medan, Indonesia; b Division of Urology, Department of Surgery, Faculty of Medicine, Universitas Sumatera Utara – Haji Adam Malik General Hospital, Medan, Indonesia; c Department of Urology, Universitas Sumatera Utara Hospital, Universitas Sumatera Utara, Medan, Indonesia

**Keywords:** Pigtail suture stents, double J stent, ureteral stent symptoms, endourology, urolithiasis

## Abstract

**Introduction:**

Pigtail suture stent (PSS) was introduced to reduce the occurrence of ureteral stent symptoms that often occur after placement of DJ stent for most endourology procedures. This study aims to conduct a review and meta-analysis to compare the occurrence of ureteral stent symptoms between PSS and conventional DJ stent using the validated Ureteral Stent Symptom Questionnaire (USSQ).

**Methods:**

Literature search was done under the PRISMA guidelines. Eligible studies were included, data were extracted, and pooled analyses were made according to each domain of the Ureteral Stent Symptom Questionnaire (USSQ), namely Urinary Index score, Pain Index score, General Health Index score, Work Performance Score, and Sexual Matters. Pooled effect estimates were generated as Mean difference and their 95%CI, statistical significance was defined with P-value < 0.05, and heterogeneity was examined using I^2^ analyses. Sensitivity analyses were done to omit confounding.

**Results:**

A total of four eligible studies were identified and included in the meta-analysis. A total of 314 patients were included across two RCTs and two prospective cohort studies. Among the USSQ domain, Urinary index (MD = −10.11; 95% CI: −15.24, −4.98; *p* = 0.0001), pain index control (MD = −8.05; 95% CI: −15.63, −0.48; *p* = 0.04), and work performance (MD = −1.90; 95% CI: −2.34, −1.46; *p* < 0.00001) were found to be improved better in PSS compared to conventional DJ stent, while general health index and sexual matter were comparable. Sensitivity analyses were conducted, and no studies were omitted.

**Conclusion:**

Ureteral stent-related symptoms were significantly better in the PSS compared to conventional DJ stent post-endoscopic surgeries in urology, in terms of urinary and pain index, as well as the work performance domain of USSQ. Limitations include the small number of studies, heterogeneity in study design, and differences in PSS type used.

## Introduction

Ureteral stents play a vital role in urological surgery [1]. They facilitate the passage of stone fragments post-treatment and help prevent obstructions or the delayed onset of ureteral strictures [1,2]. A stent can be utilized to evacuate a blocked and infected kidney in emergency situations or can be implanted prior to surgery to facilitate the widening of the ureter in preparation for surgical procedures, such as those involving advanced cervical cancer, where tumor persistence leads to obstructive uropathy [1–3]. Urinary stents play a crucial role in various situations that require urine drainage. Acute renal colic and obstructive pyelonephritis represent urgent indications. During post-endoscopic procedures, safety considerations include ureteral edema or perforation, steinstrasse, a history of renal failure, and the presence of a single or transplant kidney [4].

Although stent pain varies greatly from patient to patient, it is thought to impact more than 80% of individuals. Numerous studies in the literature list the symptoms associated with ureteral stents along with their estimated incidence. These symptoms include dysuria (40%), incomplete emptying (76%), flank (19–32%) and suprapubic pain (30%), incontinence, and hematuria (25%) as well as irritative voiding symptoms such as frequency (50–60%), urgency (57–60%), dysuria (40%), and incomplete emptying (76%) [5–11]. The primary objective of an ureteral stent is to facilitate urine flow and reduce the risk of urinary tract obstructions, both immediate and long-term. One major concern with stents is their tolerability [2]. Stents can give rise to problems such as encrustation and bacterial growth, potentially leading to symptomatic urinary tract infections [12]. Stent-related symptoms (SRS) can be caused by the stent itself or problems like migration or displacement. They can also be caused by how rigid or flexible the material used to make the stent is [13,14].

Two commonly employed types of ureteral stents are the pigtail sutured stent (PSS) and the conventional double-J (DJ) stent [2,15]. While both serve the fundamental purpose of ensuring urine flow, they differ in design and mechanism [15]. The pigtail sutured stent features a unique coiled design with suture fixation, while the conventional DJ stent is characterized by its traditional double-pigtail shape [15]. This comparison aims to examine the advantages and disadvantages of these two stent types, shedding light on their respective clinical utility, patient outcomes, and potential complications [15]. It is hypothesized that PSS may reduce mechanical stimulation of the bladder mucosa and improve patient comfort due to its distal suture design.

Before 2003, research did not typically use a validated questionnaire to document patient-reported symptoms related to stents. This changed with the development of the Ureteral Stent Symptom Questionnaire (USSQ) by Joshi et al. [16] The USSQ allows for the objective measurement of patient-reported stent-related symptoms and includes six domains: urinary symptoms (Urinary Index score), limitations in work performance (Work Performance score), stent-related pain (Pain Index score), overall general health (General Health Index score), a section related to sexual matters (Sexual Matters), and additional concerns (Additional Problems) [16].

Our aim is to conduct a comparative analysis between pigtail sutured ureteral stents and conventional double-J (DJ) stents in terms of their effectiveness and patient outcomes in urological procedures, with a focus on USSQ patient-reported symptoms.

## Materials and methods

To ensure this study was conducted in accordance with best practices, we used Preferred Reporting Items for Systematic Reviews and Meta-Analyses (PRISMA) guidelines [17]. In addition, the selected studies were identified using the PICO (Population, Intervention, Comparison, and Outcome; Table 1). The authors did not conduct any new human or animal experiments for this article and relied solely on previously published research; therefore, we did not require ethics committee approval and informed consent for this research. This review was not registered in PROSPERO or another protocol registry, which is acknowledged as a limitation.

**Table 1.** PICO approach.

Population	Patients with a history of stent usage
Intervention	Pigtail suture stent
Comparison	Conventional DJ stent
Outcome	Urinary Index score Pain Index score General Health Index score Work Performance Score Sexual Matters

### Search strategy and study selection

To conduct a comprehensive search, an extensive exploration was conducted on five databases, namely PubMed, Cochrane, Scopus, EMBASE, and Science Direct, which was conducted through 15 December 2023. The search terms employed are as follows: ‘Pigtail sutured stent’, ‘DJ Stent’, ‘Conventional Stent’, ‘USSQ’, ‘Ureteral Stent Symptoms’. Information about the keywords used in each database can be seen in Table 2. Only human studies published within the last 20 years were considered for selection. Only full-text, English-language studies were included. Grey literature and conference abstracts were excluded. Additionally, the references of all studies that met the inclusion criteria were thoroughly examined, and any discrepancies during screening were resolved through discussion between reviewers. This research exclusively examines full-text studies that compare patients with a history of usage of pigtail suture stent or conventional DJ stent in the correlation with the ureteral stent symptoms developed.

**Table 2.** Keywords used in each database.

Databases	Keywords
PubMed	stent[MeSH Terms] OR ‘DJ stent’ OR ‘Conventional stent’) AND (‘Pigtail suture stent’ OR SPSS OR PSS) AND (‘Ureteral Stent Symptoms’ OR USSQ)
Cochrane	stent[MeSH Terms] OR ‘DJ stent’ OR ‘Conventional stent’) AND (‘Pigtail suture stent’ OR SPSS OR PSS) AND (‘Ureteral Stent Symptoms’ OR USSQ)
Scopus	(‘Pigtail sutured stent’) AND (‘DJ Stent’ OR ‘Conventional Stent’) AND (‘USSQ’ OR ‘Ureteral Stent Symptoms’)
EMBASE	(‘Pigtail sutured stent’) AND (‘DJ Stent’ OR ‘Conventional Stent’) AND (‘USSQ’ OR ‘Ureteral Stent Symptoms’)
ScienceDirect	(‘Pigtail sutured stent’) AND (‘DJ Stent’ OR ‘Conventional Stent’) AND (‘USSQ’ OR ‘Ureteral Stent Symptoms’)

### Study eligibility

This review exclusively included studies that met specific inclusion criteria, focusing on patients with a history of using ureteral stents, either with pigtail suture stent or conventional DJ stent. The criteria required that the studies must make a comparison between the PSS or conventional DJ stent and must be original research articles reporting outcomes based on USSQ, Urinary Symptoms, Pain, General Health, Work Performance, Sexual Matters Studies that were non-comparative, lacked full-text availability, did not separately report outcomes, or were published before 2002 were excluded from the analysis.

The risk of bias analysis was conducted using the Cochrane RoB 2 Tool for RCT studies and the Newcastle Ottawa Scale (NOS) for prospective cohort studies. The Cochrane RoB2 Tool assessed the studies on their randomization process, deviations from intended interventions, missing outcomes, measurement of the outcome, and selection of reported results. Meanwhile, the Newcastle Ottawa Scale assesses the selection bias, comparability bias, and outcome bias. The results will then be pictured into tables, with RoB2 output as low risk, some concerns, or high risk studies, and NOS output as good-quality, fair-quality, or low-quality studies.

### Screening

Two independent reviewers were responsible for the selection and inclusion of studies in this review. Duplicate records were removed using EndNote X9 and screened based on titles/abstracts. The eligibility of full-text papers was then examined, and studies that matched were added to this study.

### Statistical analysis

The selected studies for this meta-analysis were analyzed using The Cochrane Collaboration Review Manager (RevMan Version 5.4, The Cochrane Collaboration, 2020). Continuous variables were calculated using mean difference (MD) with 95% CI. The outcomes evaluated for continuous variables were Urinary Index score, Pain Index score, General Health Index score, Work Performance Score, and Sexual Matters. A p-value of less than 0.05 was termed as statistically significant. I^2^ serves as a measure of heterogeneity and ranges from 0% to 100%. When I^2^ is less than 25%, it indicates low heterogeneity. A value of 50% suggests moderate heterogeneity, and 75% suggests high heterogeneity. Depending on the level of heterogeneity, either the fixed effect model or the random effects model was used to calculate the pooled effect size. The fixed effect model was used when there was low heterogeneity, while the random effects model was used when there was significant heterogeneity.

## Results

**Figure 1.**
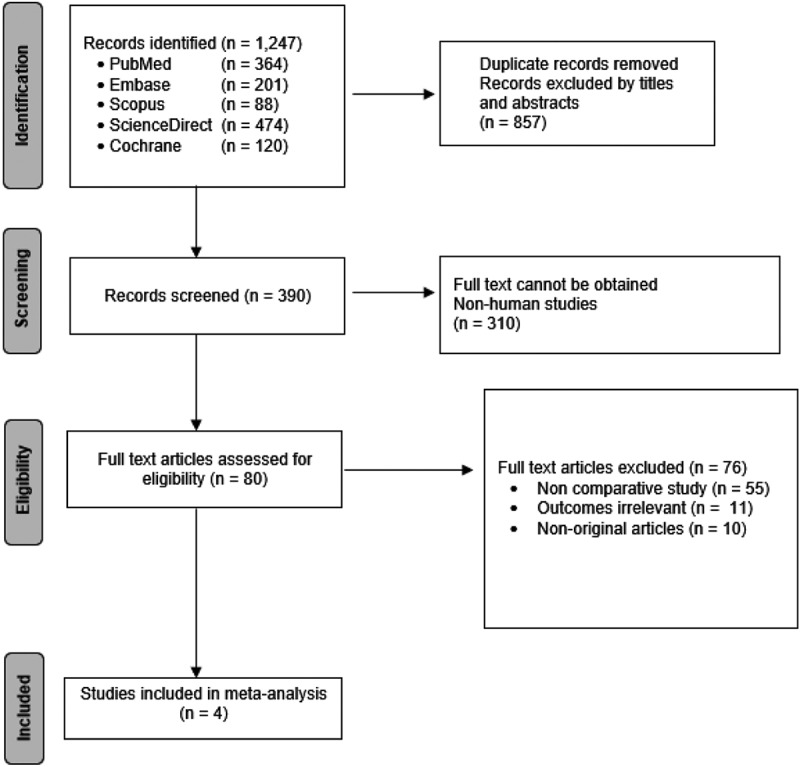
PRISMA flow diagram.

The Initial search from all databases yielded 1247 studies, with all duplicates removed using the EndNote X9 application. 76 full-text articles were excluded after screening for various reasons. Following duplicate removal and titles or abstracts screening, 80 studies were evaluated for eligibility. Consequently, four studies [18–21] were included in the meta-analysis after exclusion (Figure 1).

### Study characteristics

Out of the four studies considered, two were randomized controlled trials, and two were prospective cohort studies. There were 314 patients in the meta-analysis, with PSS performed in 180 patients (57%) and DJ Stent performed in 134 patients (43%). Studies originated from Italy, Singapore, Turkey, and France. Overall, two RCT and two cohort studies are included as shown in Table 3.

**Table 3.** Characteristics of the included studies.

Study	Study design	Country	Group	Sample Size	Age (Mean±SD)	M:F	Stent indication (Stone location)	Procedure
Bosio et al. 2021 [19]	RCT	Italy	PSS	39	53 (45–65)	27:12	Kidney: Ureter (29:10)	N/A
			DJ	39	57 (47–68)	29:10	Kidney:Ureter (30:9)	N/A
Vogt et al. 2015 [18]	Cohort	France	PSS	55	60 ± 15	34:21	Proximal stone:Distal stone:UPV junction:utereral stricture (35:11:2:7)	Stenting:SWL:Alkalization:SWL and ureteroscopy:ureteroscopy (13:6:2:12:22)
			DJ	10	53.6 ± 13.1	7:3	Distal stone (10)	Ureteroscopy (10)
Lim et al. 2022 [20]	Cohort	Singapore	PSS	21	43 (30–56)	18:3	Kidney:ureter (8:16)	URS:fURS:Cytosco±±py (13:6:2)
			DJ	20	49 (28–73)	18:2	Kidney:Ureter (1:19)	URS:fURS:Cytoscopy (17:2:1)
Bostanci et al. 2006 [21]	RCT	Turkey	PSS	65	38.63 ± 11.90	42:23	Upper:middle:lower ureter (9:18:38)	N/A
			DJ	65	37.08 ± 10.33	46:19	Upper:middle:lower ureter (10:20:35)	N/A

### Risk of bias

**Figure 2.**
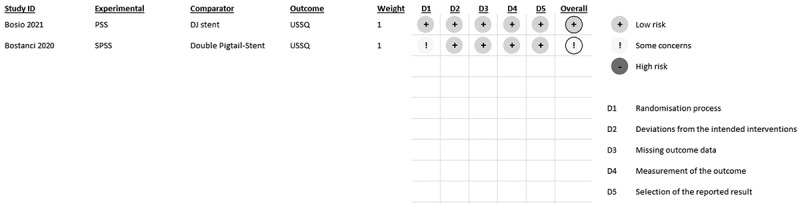
RoB 2.

Out of the four studies considered, two were analyzed using the Cochrane RoB 2 Tool for RCT Studies. One of the studies was low risk, while others had some concerns. The other two studies were analyzed using the Newcastle Ottawa Scale (NOS) for prospective cohort studies, both of them were of good quality. The risk of bias analysis results are shown in Table 4 and Figure 2.

**Table 4.** Newcastle Ottawa Scale.

	Selection				Comparability	Outcome			Conclusion
Studies	Representativeness of the exposed cohor	election of the non-exposed cohort	Ascertainment of exposure	Demonstration that outcome of interest was not present at start of study	Comparability of cohorts on the basis of the design or analysis controlled for confounders	Assessment of outcome	Was follow-up long enough for outcomes to occur	Adequacy of follow-up of cohorts	
Vogt et al. 2015 [18]	*		*	*	*	*	*	*	Good quality
Lim et al. 2021 [19]	*	*	*	*	*	*	*	*	Good quality

### Urinary Index score

**Figure 3.**
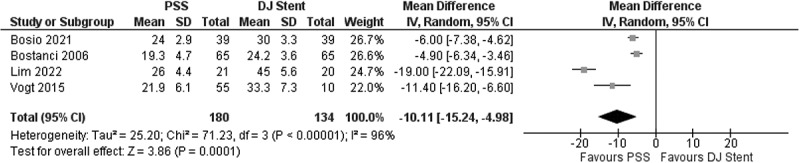
Urinary Index score comparison between PSS and conventional DJ stent.

Our study found that the Urinary Index Score domain in the PSS group was significantly better in the PSS group compared to the conventional DJ Stent group with MD = −10.11; 95% CI: −15.24, −4.98; *p* = 0.0001 (Figure 3).

### Pain index score

**Figure 4.**
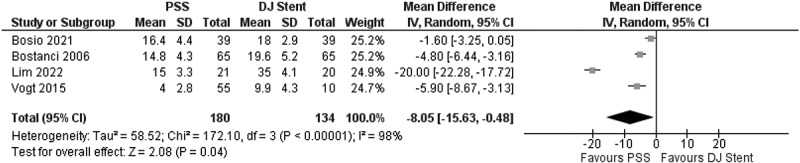
Pain Index score comparison between PSS and conventional DJ stent.

A total of four studies were included. The overall analysis on Pain Index Score showed significantly better results regarding pain symptoms in PSS stented patients compared to conventional patients (MD = −8.05; 95% CI: −15.63, −0.48; *p* = 0.04). Less pain is generated from the usage of PSS compared to conventional DJ Stent usage for patients (Figure 4).

### Work performance score

**Figure 5.**
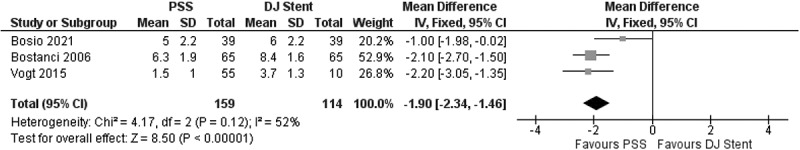
Work performance score comparison between PSS and conventional DJ stent.

From the three included studies with a total of 273 included patients, we compared PSS and DJ Stent based on the work performance score for each study. From our results, we found that PSS has shown significant favorability in besting the DJ Stent on these scoring criteria (MD = −1.90; 95% CI: −2.34, −1.46; *p* < 0.00001) (Figure 5).

### Other findings

A total of two studies were included for the general health index score and sexual matters analysis. The overall analysis showed no significant results favoring any group. This result was based on 208 patients in total from two different stent groups (MD = −1.65; 95% CI: −3.55, −6.84; *p* = 0.53). Based on two studies included in the analysis for Sexual Matters, we found out that PSS and DJ stent were comparable (MD = −0.10; 95% CI: −0.40, 0.19; *p* = 0.5). No significant difference was found in the Sexual Matters domain. Two studies reported complications and adverse events. But only one study showed complications which are severe hematuria (Clavien-Dindo grade I in the DJ group) and fever >38°C (Clavien-Dindo grade II, 3 PSS, and 1 DJ). Forest plots are included in the supplementary materials.

### Sensitivity analysis

Sensitivity analysis was done by omitting studies done by Bostanci, et al. due to the use of different materials of suture compared to other studies. However, no notable changes in the results were found; therefore, the study is still included in the final result. Forest plots of the sensitivity analysis are available upon request through correspondence.

## Discussion

The use of DJ stent in urological practice has been regarded as irreplaceable for comfort blanket and safety measure after most endoscopic procedures for the majority of healthcare professionals [22,23]. However, as DJ stent placement is reportedly involved with the occurrence of ureteral stent symptoms, the use of DJ stent has been brought up on debates for its necessity [22]. To overcome this adversity, PSS was developed and has been regarded as beneficial in order to overcome complaints post-surgery [18]. Several studies have reported the outcomes with a validated tool (USSQ) to quantify ureteral stent-related symptoms albeit with varying methodologies and materials used within their studies [18–21]. Our study is the first to create a comprehensive systematic review and meta-analyses of available studies on the comparison of conventional and PSS stents on ureteral stent-related symptoms that occur after placement and showed superiority of PSS in different component parameters within USSQ compared to conventional DJ stent.

Among the components of the USSQ that were investigated, the urinary index had the greatest extent of difference in effect size (MD = −10.11; 95% CI: −15.24, −4.98; *p* = 0.0001) compared to control. It should be noted that the urinary index comprised of items which are commonly found after DJ stent placement, such as LUTS (either obstructive or irritative), dysuria, hematuria, and urge incontinence [16]. The second component that was shown to be greatly reduced by PSS is the pain index, where less pain was notably reported in comparison to control (MD = −8.05; 95% CI: −15.63, −0.48; *p* = 0.04). The items comprised in the pain index of the USSQ were involving pain felt not only on bladder, external genitalia, and groin area but also on sites like flank and hypochondrium [16]. The component also assesses pain while doing physical activities and voiding. The final components of USSQ that showed statistical difference to control were work performance, albeit with modest effect size (MD = −1.90; 95% CI: −2.34, −1.46; *p* < 0.00001). It occurs that patients were affected in their work performance as portrayed by the items involving subjective thoughts on work failure, missing work, reduced working hours, inability to concentrate, functional limitation, and reduced quality of work [16]. Other components of the USSQ, the general health, and sexual matters were comparable to control, suggesting no direct effect from the replacement of conventional DJ stent with PSS.

Comparisons among currently available studies were mostly consistent [18–21]. A study done by Lim et al. that specifically investigated the benefit of PSS on Asian demographics showed better scores in urinary and pain index, especially in loin pain (possibly due to reflux) and pain at voiding [20]. Lim, et al. also reported that despite the height difference of Asian and Caucasian demography, there was no difference in outcome between the use of 8 cm or 16 cm PSS in Asian patients [20]. Other studies by Bosio, et al. [19] and Bostanci, et al. [21] both reported better scores from PSS with p-values of 0.001 and <0.001, respectively. Lim, et al. [20] in their study highlighted that the overall urinary index in the USSQ score after removal of stent was also significantly better than the control. General and sexual health are two domains that were proposed by Joshi, et al. [16] to be affected by placement of ureteral stents due to morbidity, hospitalization, and lost work hours as well as decreased self-confidence and libido associated with urinary symptoms [24]. In our study, however, there seems to be no difference between PSS and conventional DJ stent, indicating that the problem may not actually be related to the symptoms that were involving the bladder mucosa and reflux but rather psychological alteration relating to having a foreign object implanted in their bodies [24].

Before the development of PSS, studies on stent sizes, forms, and compositions have tried to investigate a way to reduce stent-related symptoms [12,25–29]. Replacement of loops to be more flexible by Joshi, et al. [30] did not show any changes. The decrease in diameter of stent by Davenport, et al. [31] also did not show any effect. The utilization of a collection of loops or thinned tails at the distal part showed inconsistent results [32,33]. Only a shorter bladder loop that does not extend throughout the bladder showed reduced symptoms compared to a longer loop [34,35]. These findings then became a pivotal point of the development for PSS, albeit the very first idea that was started by Ponsot, et al. [36] and Dauleh, et al. [37] in 1994–1995, where the lower loop was replaced by a fine, strong nylon loop.

The pigtail suture stent was developed to achieve less complaints regarding ureteral placement after most endoscopic management that may have to be kept for months, depending on their indications [18]. The placement of the conventional distal DJ stent loop within the bladder and UVJ often resulted in urinary symptoms such as pain, Irritative LUTS symptoms, and to some extent hematuria [13]. These pigtail suture stents are often comprised of an upper segment conventionally structured pigtail with a proximal loop in the pelvicalyceal system and a lower segment where the distal part of the DJ stent was replaced with sutures, generally using 0.3 Fr double surgical threads that are tailored and cut depending on the sex of the implanted [23]. The rationale for this replacement was elaborated by Mosli, et al. [38] and Vogt, et al. [23] where the distal end of the DJ stents is suggested to be involved with Stent-related symptoms and LUTS due to direct exposure to bladder mucosa and vesicoureteral reflux. Therefore, replacement may avoid lower ureteral smooth muscle spasms, local irritation to the highly neuronal trigonal mucosa, and excessive rise in intrapelvic pressure by the reflux [23,38].

Due to its direct contact with the bladder mucosa, the distal end of DJ stents has been studied as one of the primary variables determining SRS. It is also involved in the vesicoureteral reflux mechanism and lower urinary tract symptoms. In order to achieve a better-tolerated device, Vogt et al. developed a self-made PSS based on these considerations. The distal part of a DJ stent was replaced with a 0.3Fr suture that reached the bladder, avoiding a material impact on the distal part of the ureter and the bladder mucosa. In 24 patients who had a DJ stent replaced with a PSS and were severely complaining of SRS, their observational analysis revealed a decline in USSQ ratings. When compared to a traditional double J stent, a pigtail suture stent dramatically decreased stent-related symptoms, especially pain and urine symptoms [39].

After simple ureteroscopy for renal or proximal to mid-ureteral stones, our findings suggest that pigtail suture stents may offer advantages instead of a double J stent. Despite being the first meta-analysis to address the benefits of PSS compared to the conventional DJ stent, our study falls short due to the limited number of existing related publications. Only four studies were included, with different methodologies, half were RCTs while the other half were observational studies. Our study, however, is able to include about a total of 314 participants in comparison with the very small number of samples included in the individual studies, thus hopefully being able to provide more reliable results. Future high-quality RCTs with a larger number of participants would benefit this topic, as heterogeneities and bias can be avoided. Another important note is the non-identical PSS that is used by the different studies. Since there is no yet available and accessible commercial DJ stent in the market, one different PSS was noted from the study done by Bostanci, et al. where the sutures were synthetic non-absorbable silicone-coated made of polyester, which have different materials and diameters in comparison with the mostly used 0.3 Fr non-absorbable suture without coatings in other publications. To address this limitation, we tried to omit the study in our sensitivity analysis, albeit decided to eventually include it in the final version, since there is no notable difference in the pooled result of effect estimates. Despite including four studies, differences in study design, suture material, and patient populations limit generalizability.

## Conclusion

Pigtail Suture Stent would result in better ureteral stent-related symptoms in comparison with conventional DJ stent post-endoscopic surgeries in urology, especially in the urinary, pain index, and work performance domain of USSQ. However, these findings should be interpreted with caution due to the limited number of studies, the variability in study design, and differences in the PSS types used across the studies included in this review.

## Supplementary Material

Supplemental Material

## Data and materials availability

The corresponding author can be contacted to request the information and materials used.
