# Supplementary material for: Comparison of pigtail suture stent vs conventional DJ stent in ureteral stent symptom occurrences: A systematic review and meta-analysis
Source: Arab J Urol. 2025 Sep 11;24(2):99–107. doi: 10.1080/20905998.2025.2550798 (PMC13045176; doi:10.1080/20905998.2025.2550798)
Supplement: Supplemental Material [file TAJU_A_2550798_SM4794.docx]

**Supplementary Table 1.** Questionnaire results from each studies

| Study | Study design | Country | Group | Sample Size | Urinary Index Score | Pain Index  Score | General Health Index Score | Work Performance Score | Sexual Matter |
| --- | --- | --- | --- | --- | --- | --- | --- | --- | --- |
| Bosio et al.  2021 (17) | RCT | Italy | PSS | 39 | 24 + 2.9 | 16 + 4.4 | 11 + 2.2 | 5 + 2.2 | 3 + 0.7 |
|  |  |  | DJ | 39 | 30 + 3.3 | 18 + 2.9 | 12 + 2.9 | 6 + 2.2 | 3 + 0.8 |
| Vogt et al.  2015 (16) | Cohort | France | PSS | 55 | 21.9 + 6.1 | 4 + 2.8 | - | 1.5 + 1 | - |
|  |  |  | DJ | 10 | 33.3 + 7.3 | 9.9 + 4.3 | - | 3.7 + 1.3 | - |
| Lim et al.  2022 (18) | Cohort | Singapore | PSS | 21 | 26 + 4.4 | 15 + 3.3 | - | - | - |
|  |  |  | DJ | 20 | 45 + 5.6 | 35 + 4.1 | - | - | - |
| Bostanci et al.  2006 (19) | RCT | Turkey | PSS | 65 | 19.3 + 4.7 | 14.8 + 4.3 | 12.7 + 4.7 | 6.3 + 1.9 | 5.9 + 2.1 |
|  |  |  | DJ | 65 | 24.2 + 3.6 | 19.6 + 5.2 | 14.5 + 5.3 | 8.4 + 1.6 | 6.4 + 1.7 |
